# Targeting RNA Polymerase Primary σ^70^ as a Therapeutic Strategy against Methicillin-Resistant *Staphylococcus aureus* by Antisense Peptide Nucleic Acid

**DOI:** 10.1371/journal.pone.0029886

**Published:** 2012-01-10

**Authors:** Hui Bai, Guojun Sang, Yu You, Xiaoyan Xue, Ying Zhou, Zheng Hou, Jingru Meng, Xiaoxing Luo

**Affiliations:** 1 Department of Pharmacology, School of Pharmacy, Fourth Military Medical University, Xi'an, Shaanxi, China; 2 No. 451 Hospital, Xi'an, Shaanxi, China; 3 Beijing Institute of Radiation Medicine, Academy of Military Medical Sciences, Beijing, China; 4 Department of Neurosurgery, No. 309 Hospital, Beijing, China; Charité-University Medicine Berlin, Germany

## Abstract

**Background:**

Methicillin-resistant *Staphylococcus aureus* (MRSA) causes threatening infection-related mortality worldwide. Currently, spread of multi-drug resistance (MDR) MRSA limits therapeutic options and requires new approaches to “druggable” target discovery, as well as development of novel MRSA-active antibiotics. RNA polymerase primary σ^70^ (encoded by gene *rpoD*) is a highly conserved prokaryotic factor essential for transcription initiation in exponentially growing cells of diverse *S. aureus*, implying potential for antisense inhibition.

**Methodology/Principal Findings:**

By synthesizing a serial of cell penetrating peptide conjugated peptide nucleic acids (PPNAs) based on software predicted parameters and further design optimization, we identified a target sequence (234 to 243 nt) within *rpoD* mRNA conserved region 3.0 being more sensitive to antisense inhibition. A (KFF)_3_K peptide conjugated 10-mer complementary PNA (PPNA2332) was developed for potent micromolar-range growth inhibitory effects against four pathogenic *S. aureus* strains with different resistance phenotypes, including clinical vancomycin-intermediate resistance *S. aureus* and MDR-MRSA isolates. PPNA2332 showed bacteriocidal antisense effect at 3.2 fold of MIC value against MRSA/VISA Mu50, and its sequence specificity was demonstrated in that PPNA with scrambled PNA sequence (Scr PPNA2332) exhibited no growth inhibitory effect at higher concentrations. Also, PPNA2332 specifically interferes with *rpoD* mRNA, inhibiting translation of its protein product σ^70^ in a concentration-dependent manner. Full decay of mRNA and suppressed expression of σ^70^ were observed for 40 µM or 12.5 µM PPNA2332 treatment, respectively, but not for 40 µM Scr PPNA2332 treatment in pure culture of MRSA/VISA Mu50 strain. PPNA2332 (≥1 µM) essentially cleared lethal MRSA/VISA Mu50 infection in epithelial cell cultures, and eliminated viable bacterial cells in a time- and concentration- dependent manner, without showing any apparent toxicity at 10 µM.

**Conclusions:**

The present result suggested that RNAP primary σ^70^ is a very promising candidate target for developing novel antisense antibiotic to treat severe MRSA infections.

## Introduction

As a frightening “superbug”, methicillin-resistant *Staphylococcus aureus* (MRSA) has long been an overwhelming human pathogenic threat in healthcare-associated infections [1]. Its prevalence and adaptability in both community and hospital environment makes healthy patients and immune-deficient patients [2] at high risk of infection [3], [4]. Its continued pathogenicity and virulence [5], [6] causes invasive infection in bloodstream [7], essential organs, and tissues [8], [9], therefore leads to severe clinical presentations and high mortality rate [4], [10]. This is primarily due to the high incidence of methicillin-resistance that has failed almost all available antibiotics [11]. Furthermore, there has been an increase in reports of isolated MRSA strains developing multi-drug or vancomycin-(intermediate) resistance [12], [13], which exacerbated antibiotic paucity. Interventions like vigilant monitoring of antibiotic susceptibilities and judicious use of culture-directed antibiotic agents have been a long-sought endeavor, yielding limited success [14]. Meanwhile, researchers and pharmaceutical industry have been driven to discover new MRSA-active agents (i.e. new chemical derivatives or compounds with new targets [15], virulence inhibitors [16], natural products, and vaccines [17]) and combination therapies, resulting in few ideal drugs or solutions [18]. Thus, antibacterial strategies that provide timely and effective therapeutic countermeasures are urgently required for possible outbreaks of MRSA infections. Particularly, specific RNA silencing in bacteria by antisense antibacterial strategies can contribute to both aspects of the problem [19].

Antisense antibacterials are short (about 10- to 20- bases), synthetic DNA analogs that inhibit essential genes expression at mRNA level in a sequence-specific manner [20]. Thereafter, antisense inhibition leads to bacteriocidal/bacteriostatic effect or restoration of bacterial susceptibility, which depends on the function of targeted gene. Synthetic antisense oligomers, especially peptide nucleic acid (PNA) [21] and phosphorodiamidate morpholino (PMO) [22], possess favorable properties in light of antisense antibacterial application, including improved targeting specificity, binding affinity, biological stability and access to a variety of chemical modification. Meanwhile, instead of simple mixture, cell penetrating peptides (CPP) can be covalently attached/conjugated at the end of PNA or PMO chain to enhance cellular uptake of antisense oligodeoxynucelotides (AS-ODNs) without affecting Waston-Crick base paring between antisense oligomers and targeted RNAs [23]. Synthetic peptide-PNA or peptide-PMO conjugates targeting growth-essential genes have shown to inhibit bacterial growth in pure culture and in infected tissue culture, Thus, a range of functional genes have been identified as potential targets [24]. However, only a few early reports provided preliminary proof-of-principle evidence on antisense targeting of *S. aureus* genes for growth inhibitory effect (i.e. peptide-PNA targeting *fabI* [25], and *phoB*, f*mhB*, *gyrA*, plus *hmrB* [26]) or restoration of antibiotic susceptibility (i.e. liposome-capsulated phosphorothioate oligodeoxynucleotides targeting *mecA* [27]) in pure culture. Targeting resistance mechanism in MRSA relies on elucidation of subtle intracellular self-regulation among related genes, the consequences of its antisense inhibition being too complicated to predict [28]. Thus, identification of growth-essential genes for more potent antisense inhibition in *S. aureus* would aid the development of new anti-MRSA agents [29].

Bacterial DNA-dependent RNA polymerase (RNAP) is a key enzyme in transcription regulation and gene expression. Its function requires coordination of a core enzyme (comprising five subunits α_2_, β, β′ and ω) and an independent σ subunit that is reversibly recruited by core enzyme [30]. The RNAP core enzyme is responsible for transcription elongation, and different σs are in charge of transcription initiations from promoters that express genes in diverse function. The irreversible inhibition of RNAP thereby causes cell death. This has attracted much exploration for developing specific RNAP inhibitors (e.g. the rifamycins with fundamental clinical significance). The most developed σ^70^ family of σs, especially the primary σ^70^, is essential for initiating transcription of multiple genes in exponentially growth cells [31], which to our knowledge has not previously been demonstrated for antisense target validation in *S. aureus*. The primary σ^70^s are unique in structure, function and homology. The core regions of bacterial and eukaryotic RNAPs share structural and functional similarities, but the sequences of encoding genes are only partially homologous. Specifically, bacterial gene *rpoD* (encoding the primary σ^70^ of RNAP) shares the least homology in sequence with eukaryotic *rpoD*. Hence, in contrast to more conserved molecules, sequence-based drugs targeting *rpoD* products, including mRNAs, are less likely to cross react with host molecules. Most importantly, bacterial gene *rpoD* is highly conserved in identity and homologous in sequence among different pathogenic *Staphylococcus* species [32]. Such features are distinct advantages for developing narrow-spectrum anti-MRSA antisense agents [33].

In this study, by using four clinical pathogenic *S. aureus* genus with varying resistance patterns (including antibiotic sensitive, MRSA, MDR-MRSA and VISA), we report the identification of *rpoD* as a potent target for markedly bacteriocidal effect *in vitro* and *ex vivo* by antisense peptide-PNA conjugate.

## Results

### Transcript target site selection

**Figure 1:**
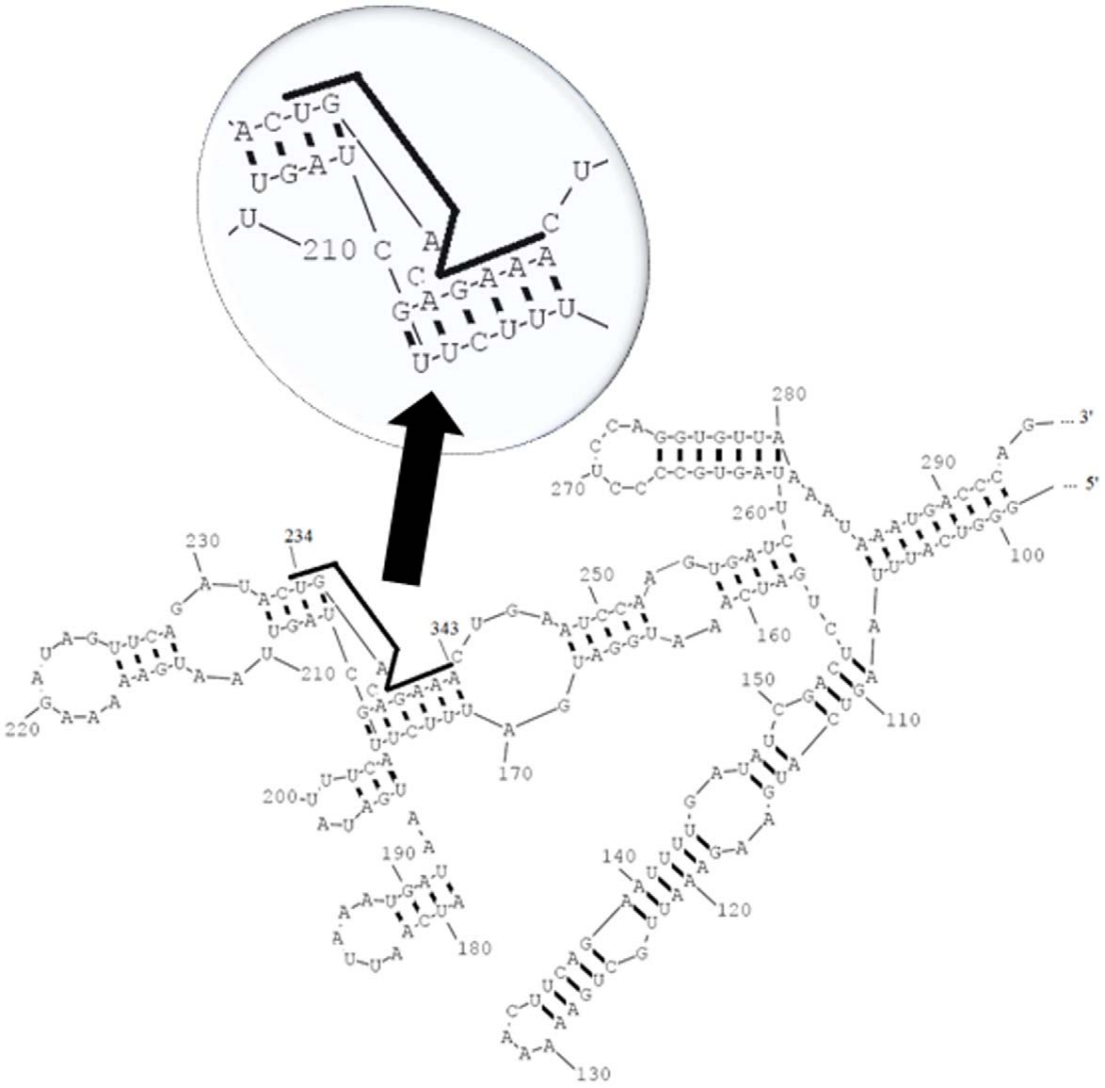
Secondary structure of *E. coli* RNA polymerase primary σ^70^ mRNA. The target region of the 10-mer peptide nucleic acid (nucleotides 234–243) is indicated by the bold line.

The *rpoD* gene encoding bacterial RNAP primary σ^70^ is highly similar in sequence among *S. aureus* species. Sequence alignment of *Staphyloccus rpoD* by Blast showed 100% gene similarity in *S. aureus*, and other *Staphyloccus* (i.e. <85% in identity for *S. epidermidis*, *S. lugdunensis*, *S. haemolyticus*, *S. pseudintermedius*, and *S. saprophyticus*,) (Table 1). Secondary structure of *rpoD* mRNA (Figure 1) was predicted by software RNA structure 4.6 and binding parameters (Table 2) were calculated by Oligo Walk plus PNALIGHT program. The combined data showed that each conserved region of *rpoD* mRNA has highly plausible sub-regions for antisense targeting. We selected total five plausible target sites for PNA synthesis (Table 2 & 3). All five mRNA regions are devoid of obvious stable secondary structures, thus they are theoretically accessible to complementary PNAs. All PNAs were covalently conjugated at the carbon terminus with peptide (KFF)_3_K (in which K is lysine and F is phenylalanine), acting as a carrier to facilitate delivery of PNA through stringent *S. aureus* cell walls [34]. The efficacy of growth inhibition was determined by measuring minimal inhibitory concentration (MIC) in liquid bacterial culture. The results are illustrated for 4 *S. aureus* strains, including *S. aureus* ATCC29213, and clinical isolates of MRSA (i.e., Mu 50, WHO-2 and XIJING) (Table 3). In all strains, PNAs complementary to the *rpoD* mRNA nucleotides (nt) encoding 36 to 47, 159 to 170, and 312 to 323 (referring to anti-*rpoD* PPNA 36, 159 and 312, respectively) were inactive at the highest concentration tested (25 µM); PNAs complementary to the *rpoD* mRNA nucleotides encoding 123 to 134 (anti-*rpoD* PPNA123) were equally active to *S. aureus* ATCC29213 and MRSA WHO-2, with a MIC value of 25 µM, whereas inactive to MRSA/VISA Mu50 and XIJING; PNAs complementary to the *rpoD* mRNA nucleotides encoding 233 to 244 (anti-*rpoD* PPNA233) were active to all 4 *S. aureus* strains, with a MIC value of 12.5, 12.5, 12.5 and 25 µM, respectively.

**Table 1:** Homologies of gene *rpoD* among *Staphylococcus* species.

Organism	GenBank	Similarity	Identity
***Staphylococcus aureus subsp. aureus***			
Mu50	BA000017.4	100%	100%
Mu3	AP009324.1	100%	100%
T0131	CP002643.1	100%	100%
Str. JKD6008	CP002120.1	100%	100%
TW20	FN433596.1	100%	100%
ED98	CP001781.1	100%	100%
JH1	CP000736.1	100%	100%
NCTC 8325	CP000253.1	100%	100%
USA300_TCH1516	CP000730.1	100%	100%
USA300_FPR3757	CP000255.1	100%	100%
ST398	AM990992.1	100%	100%
MRSA252	BX571856.1	100%	100%
MSSA476	BX571857.1	100%	99%
JKD6159	CP002114.2	100%	99%
ED133	CP001996.1	100%	99%
***Other Staphylococcus***			
*Staphylococcus epidermidis* RP62A	CP000029.1	100%	85%
ATCC 12228	AE015929.1	100%	85%
*Staphylococcus lugdunensis* HKU09-01	CP001837.1	100%	84%
N920143	FR870271.1	100%	84%
*Staphylococcus haemolyticus* JCSC1435	AP006716.1	100%	84%
*Staphylococcus carnosus subsp. carnosus* TM300	AM295250.1	100%	84%
*Staphylococcus pseudintermedius* ED99	CP002478.1	100%	83%
HKU10-03	CP002439.1	100%	83%
*Staphylococcus saprophyticus subsp. saprophyticus ATCC 15305*	AP008934.1	98%	83%

**Table 2:** Binding parameters predicted by software Oligo walk 5.0 and PNALIGHT.

PNA sequence	Conserved region of σ^70^	TargetSite^a^	Parameters^b^					
			GC(%)	overallΔG	DuplexΔG	Oligo-selfΔG	Oligo-oligoΔG	Tm(°C)^d^
								DNA:PNA
5′-gtcggatcaatt-3′	r1.1	36–47	41.7	−16.2	−16.6	0	−5.2	60.0
5′-agtttttcagca-3′	r1.2	123–133	33.3	−14.5	−14.9	0	−2.7	56.9
5′-tcatccatttga-3′	r1.2	159–170	33.3	−15.3	−15.7	0	−2.5	53.4
5′-gtttctcgtcag-3′	r3	233–244	58.3	−17.6	−18	0	0	65.1
5′-ttctcgtcag-3′	r3	233–242	40	—c	—	—	—	43.0
5′-tttctcgtca-3′	r3	234–243	40	—	—	—	—	45.9
5′-cgcccaatttct-3′	r4	312–323	50	−17.8	−18.2	0	−1.5	57.5

a Numbering from the first base of the gene *rpoD*;

b ΔG means free energy; index for each parameter: GC%≤60%, overall ΔG<−10 kcal/mol, Duplex ΔG<−25 kcal/mol, oligo-self ΔG≥−1.1 kcal/mol, oligo-oligo ΔG≥−8 kcal/mol, Tm>50°C;

c “—” means not determined;

d Calculated melting temperature in [°C] of matching PNA/DNA hybrids with no dangling ends.

**Table 3:** MIC of anti-*rpoD* peptide-PNAs for quality control and clinical strains of *Staphyloccus aureus* in M-H broth culture.

PNA designation and sequence^a^		No. of PNA bases	MIC^b^ for *S. aureus* strains (µM)			
			ATCC MRSA/VISA/MDR			
			29213	Mu 50	WHO-2	XIJING
anti-*rpoD* PPNA (without spacer between PNA and peptide)						
36 5′-gtcggatcaatt-(KFF)_3_K-3′		12	>25	>25	>25	>25
123 5′-agtttttcagca-(KFF)_3_K -3′		12	25	>25	25	>25
159 5′-tcatccatttga-(KFF)_3_K -3′		12	>25	>25	>25	>25
233 5′-gtttctcgtcag-(KFF)_3_K-3′		12	12.5	12.5	12.5	25
312 5′-cgcccaatttct-(KFF)_3_K -3′		12	>25	>25	>25	>25
anti-*rpoD* PPNA based on 233 (with spacer between PNA and peptide)						
2331 5′-(KFF)_3_K-eg^1^-ttctcgtca-3′		10	>25	>25	>25	>25
2332 5′-(KFF)_3_K-eg^1^-tttctcgtca-3′		10	12.5	12.5	12.5	12.5
2333 5′-(RXR)_4_XB-eg^1^-tttctcgtca-3′		10	6.25	6.25	6.25	6.25
Scr 2332 5′-(KFF)_3_K-eg^1^-ttttgcccat-3′		10	>40	>40	>40	>40
Scr 2333 5′-(RXR)_4_XB-eg^1^-ttttgcccat-3′		10	>25	>25	>25	>25
Free peptides KFFKFFKFFK			>60	>60	>60	>60
RXRRXRRXRRXRXB			30	30	30	30
Controls	oxacillin		0.5	>1024	512	>1024
	ceftazidine		8	1024	256	>1024
	ampicillin		1	>1024	256	>1024

a *rpoD*, RNA polymerase sigma 70; PNA, peptide nucleic acid; PPNA means peptide conjugated PNA; The PNAs are written from their N to their C terminus, and the N terminus corresponds to the 5′ end of a conventional oligonucleotide; “K” indicates lysine, F indicates phenylalanine, “X” indicates 6-aminohexanoic acid, “B” indicates β-alanine, and eg^1^ indicates glycine; Scr means PNA with a scrambled base sequence (as control);

b Minimal inhibitory concentrations (MIC) were the lowest PNA concentrations that prevented bacterial growth by visual inspection after overnight (24 h) growth from an inoculum of 10^5^ CFU/mL. “VISA” is abbreviation for vancomycin-intermediate resistance *Staphyloccus aureus*; “MRSA” is abbreviation for methicillin-resistant *Staphyloccus aureus*; XIJING means clinical MRSA isolate from patients in Fourth Military Medical University affiliated XIJING hospital.

### Anti-*rpoD* PPNA optimization

To optimize the design, we synthesized a set of antisense PPNAs based on anti-*rpoD* PPNA233 but differed in position of PNA and CPP, targeting site of PNA, PNA lengths, and CPP type (Table 3). Such design alterations should help to optimize the antimicrobial efficacy of obtained anti-*rpoD* PPNA233, and further illustrate the feasibility and sensitivity of selected target site in *S. aureus rpoD* mRNA. As shown in Table 3, the growth inhibitory effect of anti-*rpoD* PPNA2331 was abolished in all 4 *S. aureus* strains used above; anti-*rpoD* PPNA2332 and 2333 were potent, with increased activity against all 4 *S. aureus* strains. For anti-*rpoD* PPNA2332, the MICs were 12.5 µM, regardless of bacterial resistance phenotypes. Antisense specificity of anti-*rpoD* PPNA2332 was demonstrated in that PPNA with scrambled PNA sequence (Scr PPNA2332) showed no growth inhibition effect at the highest concentrations tested (40 and 25 µM, respectively). Anti-*rpoD* PPNA2333, which had the same PNA sequence of PPNA2332 but was attached to a considerably more effective peptide (RXR)_4_XB (in which R is arginine, X is 6-aminocaproic acid and B is β-alanine), showed an equal MIC value of 6.25 µM among 4 *S. aureus* strains. However, the peptide-PNAs used in this study can be membrane active, and membrane permeabilization could result in nonspecific growth inhibition. This is the exact case for peptide (RXR)_4_XB, which showed undesirable growth inhibitory effect on all 4 *S. aureus* strains at 30 µM. Although this concentration was much higher than that of the conjugate PPNA2333 (MIC = 6.25 µM), it indicated that only anti-*rpoD* PPNA 2332 provided improvements and properties that merit further evaluation.

### Bacteriocidal antisense effect of anti-*rpoD* PPNA2332

**Figure 2:**
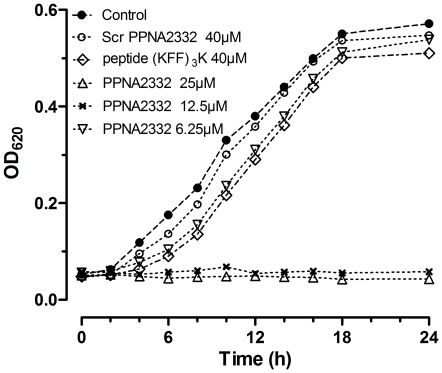
Effects of anti-*rpoD* PPNA2332 on the growth of MRSA/VISA Mu50 in pure culture. Anti-*rpoD* PPNA2332 was added to cell cultures containing 1.0×10^5^ CFU/mL MRSA/VISA Mu50 to a final concentration of 6.25, 12.5, or 25 M. Additional cell cultures were treated with free MH broth, scrambled PPNA2332 (final concentration of 40 µM), and peptide (KFF)_3_K (final concentration of 40 µM) in a volume equal to that of the PPNA2332 preparation as controls. The growth of different groups of MRSA/VISA Mu50 cells was monitored by using OD measurements. The data are shown as means for 2 samples from 2 independent tests.

**Figure 3:**
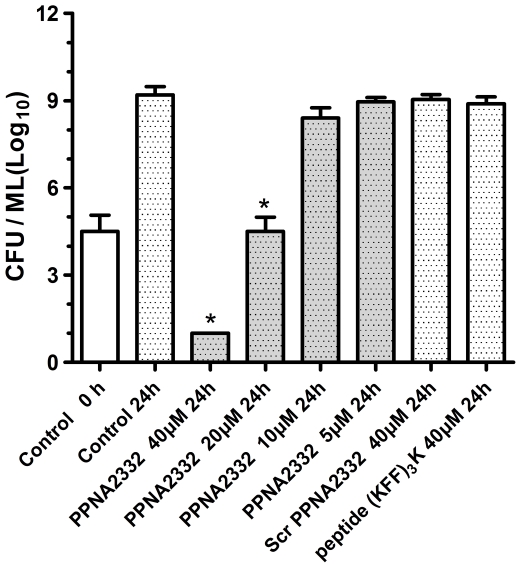
Bacteriocidal effects of anti-*rpoD* PPNA2332 on the viable cells of MRSA/VISA Mu50 in pure culture. Anti-*rpoD* PPNA2332 was added to cell cultures containing 1.0×10^5^ CFU/mL MRSA/VISA Mu50 to a final concentration of 5, 10, 20, or 40 µM. Additional cell cultures were treated with free MH broth, scrambled PPNA2332 (final concentration of 40 µM), and peptide (KFF)_3_K (final concentration of 40 µM) in a volume equal to that of the PPNA2332 preparation as controls. Aliquots of each culture were collected at 0 h and 24 h, diluted, and inoculated onto solid MH agar. The number of CFU was calculated from the number of colonies growing on plates. The 24 h counts for 40 µM PPNA2332 were 0 CFUs/mL. The data are shown as means ± SD for 2 samples from 2 independent test. *, P<0.01 for comparison to control values.

The growth inhibitory effect of anti-*rpoD* PPNA2332 on bacterial cells were further examined by assessing cell growth and viability. In pure culture of MRSA Mu 50, we observed a time- and concentration- dependent inhibition of *S. aureus* growth in MH broth with PPNA2332. Scrambled PPNA2332 and control peptide (KFF)_3_K did not show any significant growth inhibitory effect at higher concentrations (Figure 2). There was no appreciable difference in growth between the cultures incubated with the Scr PPNA2332 or peptide (KFF)_3_K and those incubated without PPNA2332. All cultures grown without PPNA2332 increased by <4 log after 24 h of growth (1×10^5^ CFUs/mL at 0 h) (Figure 3). However, PPNA2332 against *rpoD* mRNA was bactericidal in culture at ≥20 µM concentrations, with a >4 log reduction in CFUs at 24 h, compared with the culture at 0 h (P<0.01). Full elimination of live bacterial cells was observed for PPNA2332 at 40 µM when plated undiluted (limit of detection, 10 CFUs/mL). There were no difference in numerated colonies between all samples from cultures treated with Scr PPNA2332 or control peptide (KFF)_3_K and that of growth control. These results suggested that anti-*rpoD* PPNA2332 exerts a concentration-dependent bacteriocidal antisense effect.

### Effect of anti-*rpoD* PPNA2332 on corresponding gene transcripts and protein expression

**Figure 4:**
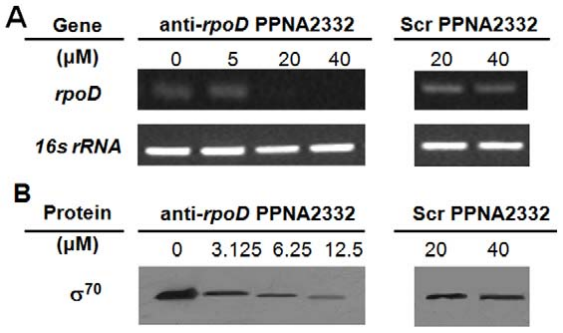
Specific inhibition of RNAP primary σ^70^ gene transcript and protein expression by anti-*rpoD* PPNA 2332. Triplicate bacterial cultures were grown for 18 h in 100 µL of MH broth in the presence of anti-*rpoD* PPNA2332 and scrambled PPNA2332 at different concentrations with MRSA/VISA Mu50 cells, respectively. The anti-*rpoD* PPNA2332 was added once at the start of the growth period. After 18 h of growth, total RNA and protein was isolated from treated and untreated cultures. (A) Cellular levels of σ^70^ RNA were determined by RT-PCR. The reduction in the amount of RT-PCR product corresponding to gene *rpoD* in cells treated with anti-*rpoD* PPNA2332 at different concentrations was determined as the product specific for *rpoD* RNA relative to products for *16S rRNA*. (B) Expressions of σ^70^ protein in different treatment were analyzed by western blotting and were quantitated densitometrically.

The reduction in viable bacterial cells we measured could be caused by a decay of specific transcripts of gene *rpoD* through antisense mechanism and subsequent decreased expression of protein σ^70^. To verify this possibility, we performed Reverse Transcription Polymerase Chain Reaction (RT-PCR) and western blotting for each individual transcript with total RNA and protein isolated from 18-hour MRSA/VISA Mu50 cultures upon inhibition of anti-*rpoD* PPNA 2332 at different concentrations. We observed that the levels of *rpoD* mRNA were greatly diminished in a concentration-dependent manner in treated cultures compared with untreated cultures (Figure 4A). Further, full suppression of *rpoD* mRNA was observed for PPNA 2332 treatment at 40 µM, which was probably due to steric blockage on top of mRNA decay effects. The level of the unrelated, constitutively expressed 16S rRNA gene transcript, assayed as a control RNA, did not change under any of the culture conditions. Again, the sequence specificity of the antisense gene knock-down effect was evaluated in each bacterial culture treated with Scr PPNA2332, and the expression of *rpoD* at mRNA level was not affected by the control condition at doses up to 40 µM (Figure 4A). To ascertain whether the antisense inhibition of *rpoD* at mRNA level could lead to decreased expression of its protein product, the expression of σ^70^ was tested. Interestingly, inhibiting the *rpoD* mRNA by PPNA2332 led to decreased expression of σ^70^ in a concentration-dependent manner (Figure 4B). But the Scr PPNA2332 treatment showed no effect on the expression of σ^70^. These results demonstrated that RNAP primary σ^70^ was crucial for sustaining bacteria surviving, and knock-down of *rpoD* by PPNA2332 induced transcript decay and unsuccessful translation of transcripts.

### Protective effect of anti-*rpoD* PPNA2332 on human cultured cells from single MRSA infection

**Figure 5:**
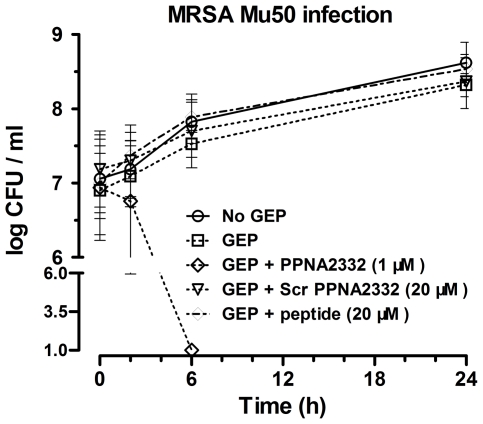
Protective effect of anti-*rpoD* PPNA2332 on epithelial cell culture infected with noninvasive single MRSA/VISA Mu50 infection. The symbols represent time points of cell harvest (2, 6, and 24 h) and are displayed as the mean number of colony-forming units (CFUs) per milliliter. Error bar represents SD. The 6 h counts for 1 µM PPNA2332 were 0 CFUs/mL. For all observations in co-cultures (GEP+MRSA/VISA Mu50) treated, with the limit of detection indicated as 10 CFUs/mL. Scr PPNA2332, Scrambled sequence control PPNA2332; GEP, gastric mucosa originated epithelial cell.

To examine the antibacterial potential of anti-*rpoD* PPNA2332 in the presence of eukaryotic cells as well as to evaluate its possible toxicity, we tested it against single clinical MRSA/VISA strain Mu50 grown in gastric mucosa originated epithelial cell (GEP) culture medium. In this medium, which is likely to be more representative of *in vivo* application, PPNA2332 was >10-fold more potent (MIC = 1 µM) than in MH broth. Furthermore, epithelial cell cultures were artificially infected with 10^7^ CFU/mL of invasive MRSA/VISA Mu50 cells, and anti-*rpoD* PPNA2332, Scr PPNA2332, and control peptide (KFF)_3_K were added immediately post-infection or tissue culture medium only. This system can be viewed as a very simple model for the growth of an extracellular pathogen in a host. Bacterial CFUs from each condition were measured at 2, 6, and 24 h after infection. MRSA/VISA Mu50 grew 2 log in 24 h in the presence of medium alone (Figure 5). At 2 h after infection, PPNA2332 in infected GEP had reduced bacterial CFUs by 0.65 log (Figure 5). Further reduction of CFUs was continued over time, and by 6 h PPNA2332 caused fully elimination of CFUs, compared with infected GEP without PPNA treatment. The difference in killing was most pronounced after 24 h, when there was a 7.45 log difference (P<0.01) in CFUs between bacterial cells in GEP and the addition of PPNA2332 (Figure 5). The addition of the controls (Scr PPNA or peptide) had no appreciable effect on antisense killing of bacterial cells, compared with bacterial cells grown in GEP alone.

**Figure 6:**
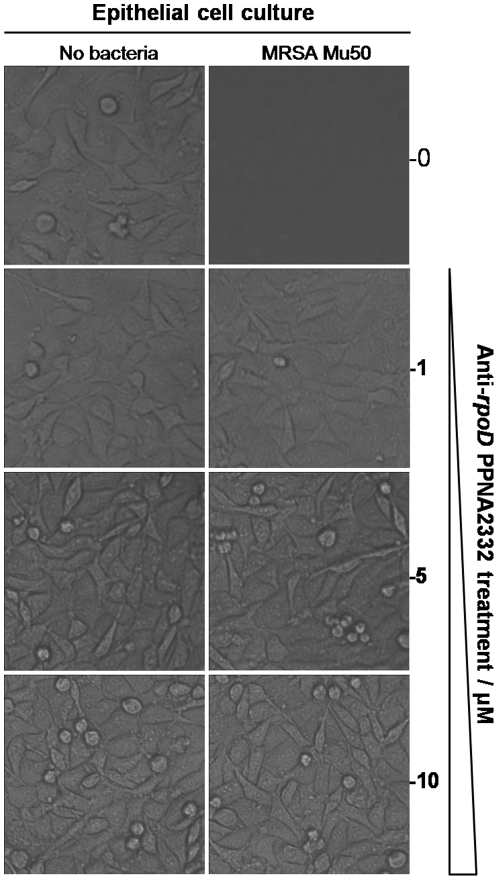
Light micrographs of epithelial cell cultures. The images show epithelial cell cultures grown in DMEM/HIGH Glucose, 10% FCS. The column at left shows epithelial cell cultures without added MRSA/VISA Mu50; the right column shows epithelial cell cultures that were inoculated with MRSA/VISA Mu50. The top panels of each column show cultures not treated with PPNA, and the rows below show cultures treated with increasing amounts of anti-*rpoD* PPNA2332 (1, 5, and 10 µM). Magnification, ×100.

These results were confirmed by microscopic examination of the cultures, which showed that anti-*rpoD* PPNA2332 treated GEP cultures appeared the same as uninfected cultures (Figure 6). All infected controls appeared the same, independent of treatment, and showed few GEP cells but massive amounts of bacteria, which made the cultures appear turbid. Results obtained using Scr PPNA2332 and peptide (KFF)_3_K were similar to those obtained in MRSA/VISA Mu50 cells in GEP culture only (data not shown).

## Discussion

Rapid evolution of resistance genes developing among Gram-positive pathogens has unfortunately exceeded our ability to develop new antimicrobials [35]. The methicillin-resistance, vancomycin-intermediate resistance or MDR- *S. aureus* present formidable challenges for traditional antibiotic discovery [36]. Novel antisense antibacterial strategies have been introduced and can efficiently provide potential drugs to combat emerging or re-emerging pathogens.

The conserved bacterial DNA-dependent RNA Polymerase (RNAP) is the target of rifamycins and is of great interest as a potential target for developing antisense bacteriocidal agents. Thus, we set out to target *rpoD* (encoding RNAP primary σ^70^) in *S. aureus* by antisense strategy, assuming it was essential for survival of pathogenic MRSA. By using peptide-PNA conjugates, our results suggest that the 234–243 nt of *rpoD* mRNA (in conserved region 3) is most sensitive to antisense inhibition. An optimized (RXR)_4_XB conjugated PNA complementary to this sequence exerted by far the most potent (MIC = 6.25 µM) antisense growth inhibitory effect against clinical isolates of pathogenic MRSA *in vitro*. Our study also showed that antisense targeting of *rpoD* by PPNA2332 could significantly inhibit growth of *S. aureus* genus of different resistance phenotypes. The effect of PPNA2332 was sequence specific, because a scrambled-sequence PPNA did not inhibit growth of *S. aureus* genus, nor did incubation with the peptide (KFF)_3_K alone. *In vitro* gene-specific bacteriocidal activity was also seen in clinical isolate of MRSA, which implied that targeting *rpoD* by high concentration of PPNA would lead to complete decay of *rpoD* mRNA and finally concomitant cell death. More importantly, micromolar PPNA2332 treatment was able to significantly rescue 100% of MRSA-infected epithelial cells, with full elimination of MRSA cells in co-culture medium. This highly implied the therapeutic potential of PPNA2332 in the treatment of MRSA infections by targeting *rpoD*.

A challenging aspect of identifying essential genes in bacteria for antisense inhibition mainly involves efforts to locate the exact targeting site within gene bases for realizing potent and specific antisense inhibitory effect of complementary AS-ODNs. Proof-of-principle evidence from previous study on identifying genes essential for growth in *S. aureus* was first offered by Nekhotiaeva et al [26]. They have used four endogenous genes in *S. aureus* RN4220, including *phoB* (encoding alkaline phosphatase), *fmhB* (involved in cell wall biosynthesis), *gyrA* (involved in DNA replication), and *hmrB* (an ortholog of the *E. coli acpP* gene and highly sensitive to antisense inhibition in *E. coli*). And they tested the bacterial growth inhibition efficacy of antisense peptide-PNAs to verify the essentiality of these genes. They only designed 2 or 3 peptide-PNAs targeting the start codon region and upstream Shine-Dalgarno (SD) region in each gene, which were previously reported for higher sensitivity to antisense inhibition. In all cases, they observed concentration-dependent growth inhibition with sequence-specificity. However, MIC values were not determined and the full-course inhibition of bacterial growth was only observed for peptide-PNA targeting *fmhB* at the concentration of 10 µM. Limited information from two other studies have shown similar MIC values (15 µM) for AS-ODNs targeting *fabI* [25] or *adk* [37] in *S. aureus* RN4220, regardless of ODN type (i.e., DNA or PNA) and delivery strategies (i.e., none or peptide mediated).

Bacterial primary σ^70^s are responsible for transcriptional initiation of multiple essential genes that play important roles on cell survival and proliferation in exponential phase. Bacteria have no bypass mechanism to retrieve the sacrifice for the inactivation of primary σ^70^s, which certainly leads to transcription failure and subsequent loss of functional proteins. Common pathogenic *S. aureus* share highly homologous sequences in gene *rpoD* (Table 1). However, little evidence has been provided to identify the accessible and sensitive regions for potent antisense inhibition. The inhibition profile from our preliminary target site selection results clearly indicates that the conserved region 3 of *rpoD* containing 233 to 244 nt sequence is of most sensitivity among five selected regions (Table 2&3). More potent growth-inhibition effect was obtained only for optimized PNA encompassing the 234 to 243 nt sequence, This further confirmed the least indispensable target sequence for effective antisense inhibition. Notably, the growth inhibition effect of the 10-mer *rpoD*-targeting PNA is through antisense mechanism, which was reflected by the concentration-dependent decay of *rpoD* mRNA transcript and decreased protein expression of σ^70^ observed for PPNA2332 treated bacterial cells rather than those treated by Scr PPNA2332.

Certain peptide-PNA and peptide-PMO oligomers targeting growth-essential genes are more bacteriocidal *in vitro* relative to molar or mass equivalent doses of ampicillin and rifampicin, and they act with gene and sequence selectivity. Potent bacteriocidal antisense effect of anti-*rpoD* PPNA2332 was highly presumable to resemble pharmacological aspects (or mode of action) of rifampicins, the famous broad-spectrum RNAP inhibitors that accumulate in bacterial cells to exert post antibiotic effect, with comparatively higher MICs than other types of antibiotics [38]. Nonetheless, rational design of peptide-PNA conjugates could enhance antibacterial potency and expand antibacterial spectrum [39]. The mechanism of uptake for such peptides and their capacity to deliver cargo molecules into cells are controversial, and there is evidence for both energy-dependent and energy-independent mechanisms [40]. Also, the uptake properties are affected by the nature of the cargo molecule. Despite these difficulties, carrier peptides appear to be one of the few options available to significantly improve effects of antisense agents in a variety of different applications. The peptide (RXR)_4_XB used in this study was previously reported as a more potent permeabilizer against *E. coli* cells than the (KFF)_3_K peptide [41], [42]. Our results suggested the (RXR)_4_XB peptide mediated more efficient delivery for clinical MRSA isolates than the (KFF)_3_K peptide, because lower MIC values were observed for (RXR)_4_XB conjugated PNA. However, (RXR)_4_XB itself showed growth inhibition effect at the concentration of 30 µM in pure culture of MRSA, indicating a small safety window. Therefore, improved peptide carriers are needed if antisense PNAs are to have broad medical applications against MRSA.

Antimicrobials can demonstrate potency *in vitro* and then fail in cell culture models or *in vivo* studies. We used isolated GEP from human donors to determine whether the addition of PPNA2332 could kill co-cultured MRSA *ex vivo* and protect human cells from lesion and death. Interestingly, more potent bacteriocidal antisense effect of PPNA2332 was demonstrated by the increase of CFUs in no treatment control cultures and the full elimination of CFUs in PPNA2332 treated culture at 24 h. This is probably due to the lower growth rate of bacterial cells in DMEM than that in MH broth. However, accumulation and sustained bacteriocidal effect of PPNAs were observed to eliminate bacteria dramatically in a time- and concentration- dependent manner with no obvious cell toxicity. Most importantly, Scr PPNA2332 showed no bacteriocidal effect, which indicated its gene-specific antisense effect in a co-cultured medium. Meanwhile, the (KFF)_3_K peptide itself exerted no growth inhibitory effect at higher concentrations in co-culture with mammalian cells, which showed the antibacterial effect of PPNA2332 was antisense mediated only.

Important challenges remain for the application of antisense technology to the treatment of gram-positive pathogens. Clearly, many issues concerning the *in vivo* activity, general bioavailability, animal and human toxicity, and pharmacokinetic behavior of peptide-PNA conjugates still need to be addressed. In this work, we firstly demonstrate that bacterial *rpoD* encoding RNAP primary σ^70^ is essential for *S. aureus* growth and is a highly promising target for developing specific antisense therapeutic agents like peptide-PNA conjugates.

## Materials and Methods

### Chemicals

All antibiotics used were purchased from the National Institute for the Control of Pharmaceutical and Biological Products (Beijing, China). All other chemicals and solvents were of analytical grade.

### Bacterial strains, CPP, and PNA

The *S. aureus* strain ATCC29213 (antibiotic sensitive, for quality control) and WHO-2 (MRSA) were obtained from the Chinese National Center for Surveillance of Antimicrobial Resistance (Beijing, China), and clinical strain MRSA XIJING (MDR) was isolated from cultures of sputum or catheter samples from patients in Xijing Hospital (Xi'an, China). MRSA/VISA Mu50 (ATCC700699) was purchased from MicroBiologics (Minnesota, USA). All strains expressed *rpoD*, which was confirmed by PCR detection (data not shown). CPPs were synthesized and purified at Genotide, Inc. (Xi'an, Shannxi, China). PNAs were synthesized and purified at Panagene, Inc (DAEHEON, Korea). peptide-conjugated PNAs were synthesized by manual coupling chemistry and purified at Panagene, Inc (DAEHEON, Korea), with the base and amino acid sequences shown in Table 3.

### PNA target site selection and anti-*rpoD* peptide-PNA (PPNA) optimization

Sequence alignment of gene *rpoD* was performed to decide the region that showed the highest sequential homology among different *S. aueus* species. Secondary structure of *rpoD* mRNA predicted by RNA structure 4.6 software. DNA:RNA and DNA:PNA paring parameters referenced by Oligo Walk 5.0 and PNALIGHT program were used to decide the site that showed best binding affinity. Building from evidence that 12-base antisense PNAs was an effective length [23], five plausible target sites located in the region that satisfied both above aspects were chosen for synthesis of complementary antisense PNAs at 12-base length. Effective target site in *rpoD* was verified by modified MIC test of PPNA in four *S. aureus* strains with different characteristics in resistance phenotype. All PNAs used are covalently conjugated with the same cell penetrating peptide (KFF)_3_K at the carbon terminus (corresponding to the 3′ end of a conventional oligonucleotide).

To further optimize the anti-*rpoD* PPNA that showed best antisense inhibitory efficacy in preliminary target site selection, de novo design in ways of (i) adding glycine spacer between PNA and CPP; (ii) shortening PNA length; and (iii) utilization of newly developed CPP (RXR)_4_XB for more efficient PNA delivery into larger scale of bacterial species, were performed. Optimal anti-*rpoD* PPNA was selected by minimal inhibitory concentration (MIC) results determined for clinical isolates of pathogenic *S. aureus* species.

### Bacterial growth assay and susceptibility testing

MICs were determined twice by the microdilution assay in sterilized 96-well polypropylene microtiter plates according to the broth microdilution guideline based on Good et al. [23]. Briefly, single colony overnight culture of subjected bacterial cells was diluted to give a final inoculation at 10^5^ CFU/mL in duplicate 100 µL Mueller Hinton (MH) broth in a low-binding 96-well microtiter plate. Stocks of PPNA, Scr PPNA and CPPs were added immediately to the indicated final concentrations. Plates were incubated at 35°C in aerobic environment for 24 h of overnight culture. MICs were the lowest PNA concentrations that prevented bacterial growth by visual inspection after overnight (24 h) growth. To verify the specific antisense effect, peptide was also conjugated to a scrambled PNA with a base sequence that was not complementary to the selected target site of *rpoD* mRNA. To control for nonspecific toxicity, the MIC of the control CPPs (KFF)_3_K and (RXR)_4_XB in all cases, was above the limit of measurement, which was 60 or 30 µM.

To determine the growth curve for MRSA/VISA Mu50 in the broth medium, cells were diluted and mixed with no PPNA, anti-*rpoD* PPNA2332 (6.25, 12.5, and 25 µM), and scrambled PPNA2332 (40 µM). The growth rate of the cells was monitored by measuring the OD values at 620 nm with a microplate reader (Bio-Rad Laboratories, Tokyo, Japan) at different time points (0, 2, 4, 6, 8, 10, 12, 14, 16, 18, and 24 h). After 24 h culture, viable cells were determined by plating 50 µL samples from each wells onto Mueller-Hinton agar plates in appropriate diluted concentrations, and numbers of colonies was counted.

### Reverse transcript (RT)-PCR

To compare σ^70^ RNA levels in inhibited and uninhibited bacterial cells, duplicate subjected MRSA/VISA Mu50 were grown as described above and treated with either no PPNA inhibitor, different concentrations of anti-*rpoD* PPNA2332, or scrambled PPNA2332 for 18 h. Cell amounts were verified by plating. Total RNA from each culture was prepared using the RNeasy Mini Kit (QIAGEN China Co. Ltd, Shanghai, China) or RNAprep pure Cell/Bacteria Kit (TIANGEN BIOTECH Co. Ltd, Beijing, China), and followed by reverse transcript using PrimeScript RT reagent Kit with DNA Eraser (TAKARA BIO INC, Kyoto, Japan). PCR was performed with the Premix Taq RT-PCR System (TAKARA BIO INC, Kyoto, Japan) according to the manufacturer's instructions. Primers specific for *rpoD* gene in subjected *S. aureus* were listed as followed. Primers specific for MRSA/VISA Mu50 *rpoD* were 5′- CAGATACTGACGAGAAA -3′ and 5′- GAATAACATACCACGAC-3′. Primers specific for MRSA/VISA Mu50 *16S rRNA* were 5′- CGTGGATAACCTACCTATAAGACT-3 and 5′-GATTCCCTACTGCTGCCTC-3′. The PCR fragment encompasses the targeting site of PPNA2332 in *rpoD* gene. Amplification was performed in a Gradient thermal cycler (BioRad laboratories Inc., Hercules, CA, USA) under the following condition: denaturation at 95°C for 3 min for the first cycle and for 30 s thereafter, annealing at 55°C for 30 s, and extension at 72°C for 40 s for 32 repetitive cycles. Final extension was at 72°C for 10 min. *16S rRNA* was used as an internal control. The PCR products were analyzed by electrophoresis on a 1% agarose gel.

### Western blotting

To compare σ^70^ expression levels in inhibited and uninhibited bacterial cells, duplicate subjected MRSA/VISA Mu50 were grown as described above and treated with either no PPNA inhibitor, different concentrations of anti-*rpoD* PPNA2332 and scrambled PPNA2332 for 18 h. Anti-*rpoD* PPNA treated bacterial cells and untreated controls were lysed using lysis buffer (Dingguo Biotech Co, Ltd, Beijing, China) containing lysozyme (100 g/mL) and PMSF (1 mM). Cell lysates were quantitated, resolved, blotted and visualized essentially as previously described. Equal amounts of protein were loaded and separated on 12% SDS-polyacrylamide gel and were then transferred to polyvinylidene difluoride membranes (Millipore Corporation, Billerica, MA, USA). The bacterial RNAP σ^70^ monoclonal antibody (Abcam, Cambridge, MA, USA) was used at dilution of 1∶1000 for overnight blotting at 4°C; the secondary horseradish peroxidase-conjugated anti-mouse antibody was used at dilution of 1∶2000.

### Epithelial cell culture, single bacterial infection, and anti-*rpoD* PPNA2332 treatment

Epithelial cells (gastric mucosa originated, a gift from Dr. Na Chai) were plated in 96-well culture dishes (Falcon, Franklin Lakes, NJ) at a concentration of 1.5×10^4^ cells/mL in a volume of 200 µL and grown at 37°C for 48 h in Dulbecco's minimal essential medium (high sugar DMEM, HyClone, Logan, Utah) supplemented with 10% fetal bovine serum (HyClone, Logan, Utah) in 95% air-5% CO_2_. An overnight respective culture of MRSA/VISA Mu50 (ATCC 700699) was diluted by high sugar DMEM with 10% fetal bovine serum for fixed-concentration experiments, and transferred to wells containing epithelial cells. The starting bacterial inoculum for single infection was 1×10^7^ CFU/mL in a 100 µL volume. Anti-*rpoD* PPNA 2332 (1, 5, and 10 µM) and scrambled PPNA2332 (20 µM) were immediately added, and the cultures were incubated at 37°C in 95% air-5% CO_2_ for 24 h. After 24 h, cultures were examined and photographed with a Nikon inverted light microscope under ×100 magnification. And culture supernatant was removed, diluted to appropriated concentration and plated on MH agar to measure viable bacterial cells.

### Statistical Analysis

Values shown in the graph are means of two or three replicates from independent experiments. Results are expressed as mean or mean ± SD where indicated. For the in vitro experiments, cell viability (in units of CFUs per milliliter) at 24 h was calculated for MRSA/VISA Mu50 and PPNA2332 treatment condition, including no treatment. The difference (in units of log count +0.5) between cultures treated with PPNA2332 and untreated cultures was analyzed by means of the paired Student t test. Values were linearly transformed by the addition of a constant, 0.5, to allow statistical testing of the log counts, as samples were completely sterilized by PPNA2332 (i.e., 0 CFUs/mL). A probability value of *P*<0.01 was considered indicative of statistical significance.
